# Supplementary material for: Mapping SP-C co-chaperone binding sites reveals molecular consequences of disease-causing mutations on protein maturation
Source: Nat Commun. 2022 Apr 5;13:1821. doi: 10.1038/s41467-022-29478-z (PMC8983781; doi:10.1038/s41467-022-29478-z)
Supplement: Supplementary file 1 — Supplementary Information [file 41467_2022_29478_MOESM1_ESM.pdf]

# Supplementary information

1. Supplementary Figures

2. Supplementary Table 1. Primers used for mutagenesis

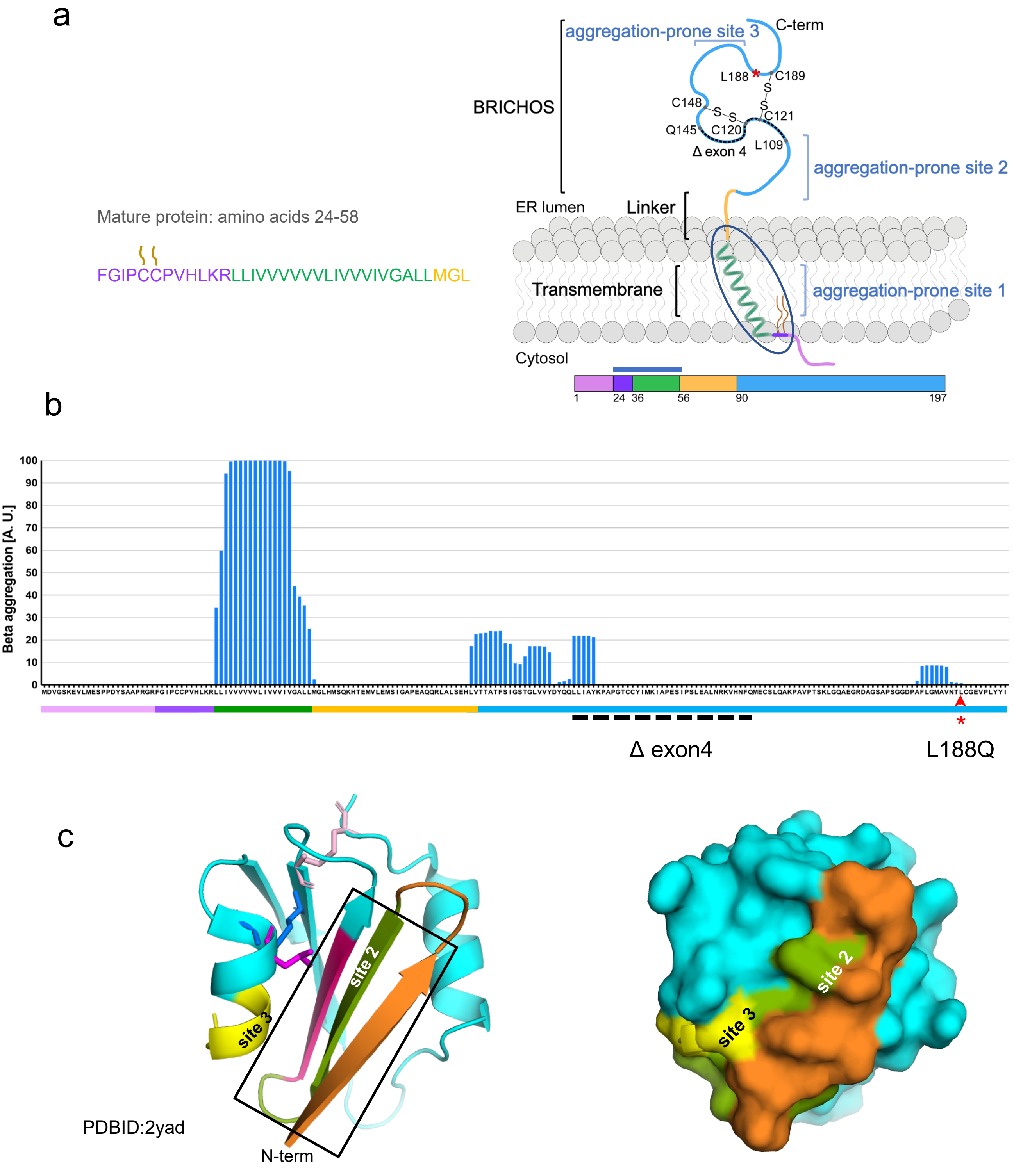


Supplementary Fig. 1

Supplementary Fig 1. Schematic with structural features of SP-C. (a) Schematic of the SP-C proprotein is shown with color-coding of the various domains with their corresponding amino acids. The mature peptide is circled on the membrane topology cartoon and indicated with a blue bar over the domain schematic below. Positions of the intramolecular disulfide bonds (C121-C189 and C120-C148) and sites of cysteine palmitoylation are identified. The region deleted in ILD-associated Δ exon 4 (L109-Q145) is portrayed with a black dotted line. Lastly, the sequences identified by the TANGO algorithm in (b) are shown on the cartoon. (b) The primary amino acid sequence of pro-SP-C was analyzed using the TANGO algorithm to detect regions that are prone to form β-strand aggregates. Three sites were identified, which are mapped on the schematic in (a). L188, which is mutated and ILD-associated mutant to L188Q, is indicated in (a) by red asterisk in (b) with both an asterisk and arrow. (c) A ribbon depiction of the BRICHOS domain with the 3 β strands that encode site 2, which are boxed, and the location of site 3 in the C-terminal α helix. The intramolecular disulfide bonds are shown. A space-filling model showing that sites 2 and 3 should be largely buried in a natively folded BRICHOS domain. The structures for the SP-C BRICHOS domain were generated using PyMOL.

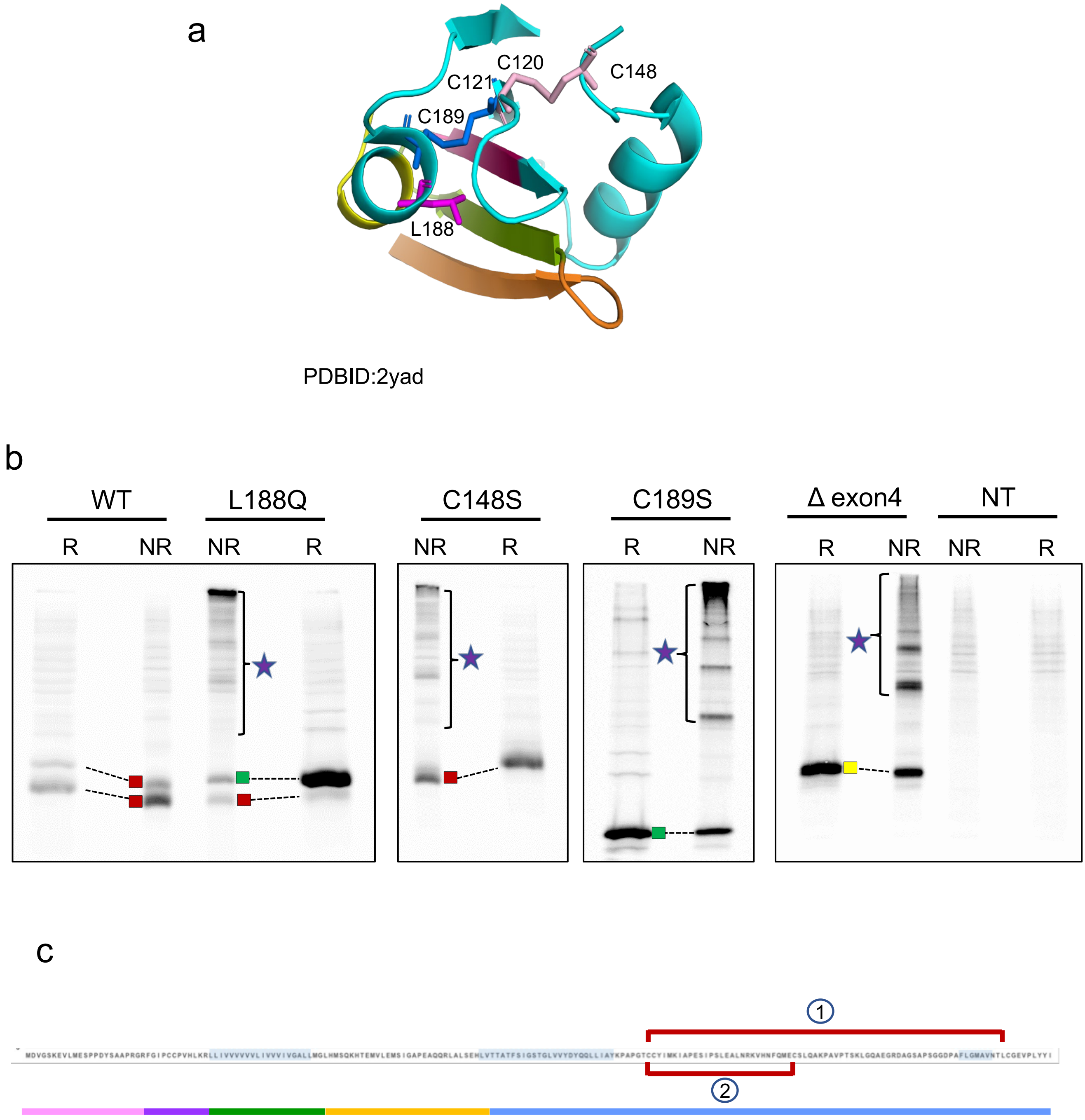


Supplementary Fig. 2

Supplementary Fig. 2. The C121 – C189 disulfide bond forms first and is more critical to proper folding and stability of the BRICHOS domain. (a) A ribbon representation of the BRICHOS domain depicting the location of the two disulfide bonds. (b) Cells expressing the indicated constructs were radiolabeled with $^{35}S$ methionine and cysteine for 3 hrs, immunoprecipitated, and analyzed as in Figure 2 (n= ≥3 independent experiments). In the case of the L188Q mutant, small amounts of fully reduced (green square) as well as partially oxidized (red square) monomers were detected, along with very large disulfide-linked oligomers that remained at the top of the gel (purple star). The Δ exon 4 mutant included a monomeric species with a single non-native disulfide bond (pink square), two predominant disulfide-bonded oligomeric species, and various other intermolecularly linked oligomers (purple star). Over half of the C148S variant existed as partially oxidized monomers (red square), and the remainder formed disulfide-linked oligomers varying in size (purple star), whereas in the case of the C189S mutant, the vast majority existed as disulfide-linked oligomers and there was no clear evidence of oxidized monomers. The dotted lines between reduced and non-reduced samples show the formation of disulfide bonds in monomers or their absence. (c) The position of the disulfide bonds and their order of formation is indicated on the linear sequence of the SP-C protein. Source data are provided as a Source Data file.

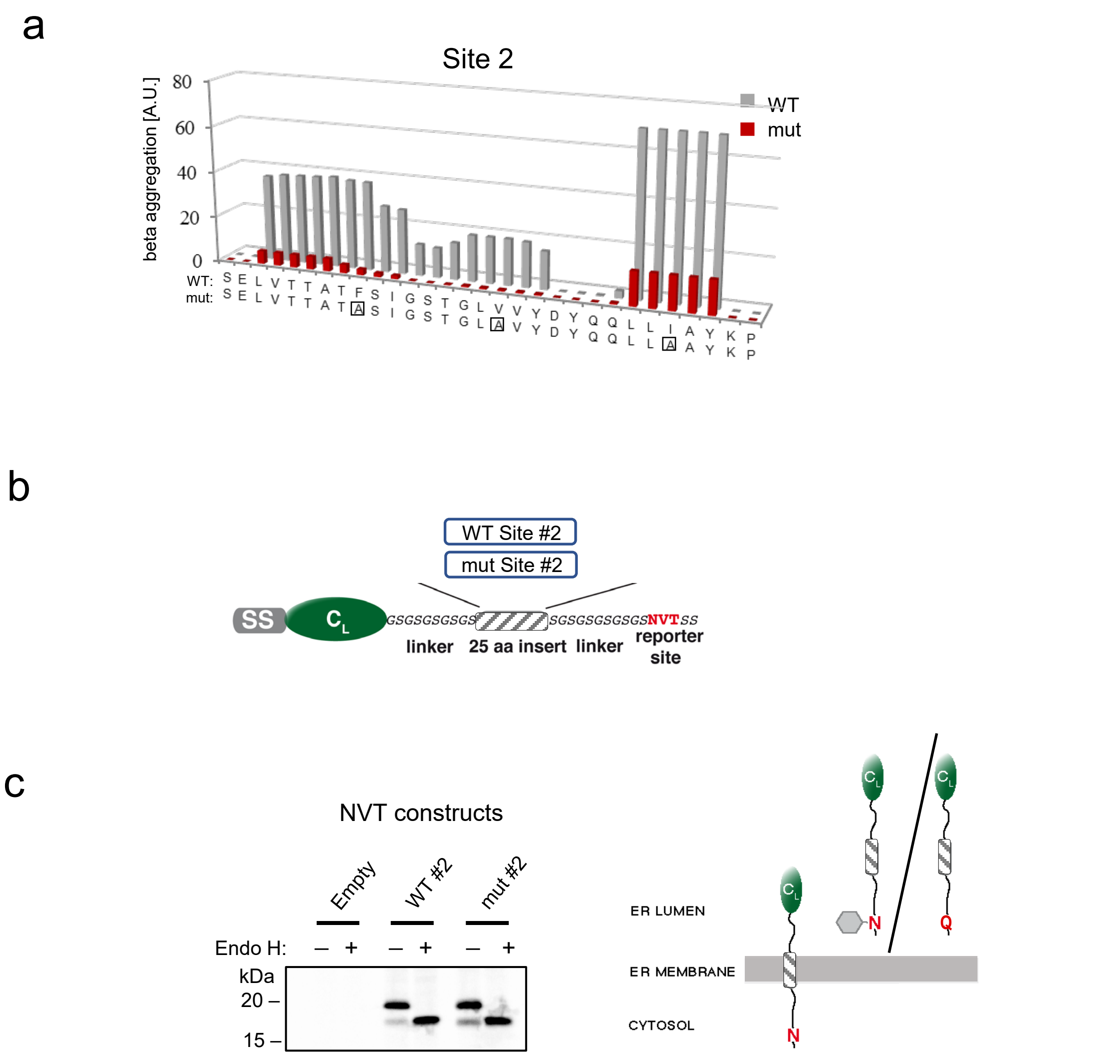


Supplementary Fig. 3

d

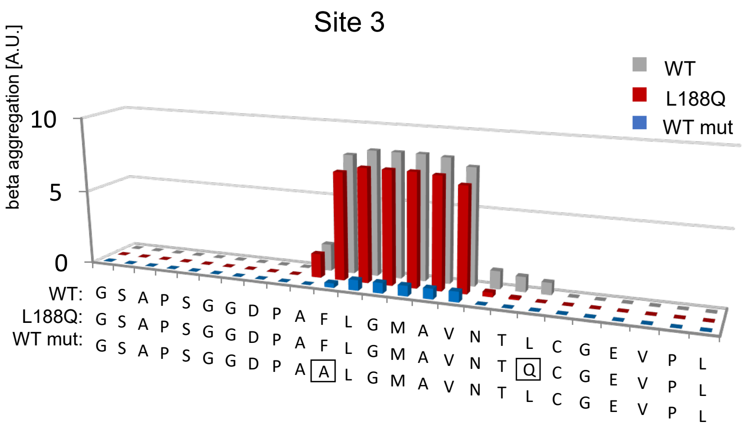


e

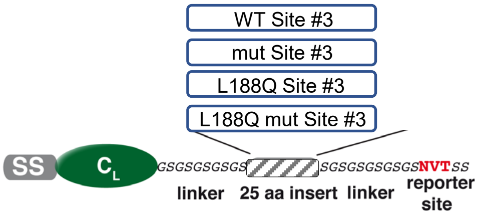


f

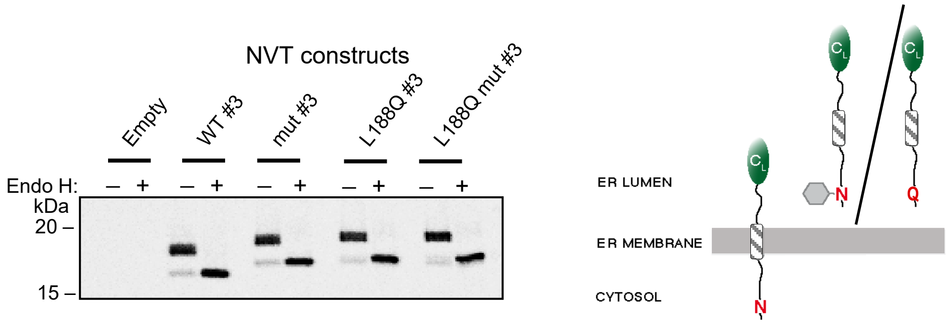


Supplementary Fig. 3

Supplementary Fig. 3. Assessment of co-chaperone binding to sites 2 and 3. (a, d) The TANGO algorithm was used to predict β-aggregation propensity of sequences, and mutations were chosen that decreased aggregation propensities. Sequences are indicated below the graph and mutated residues are boxed. For site 2, two additional amino acids are depicted at the N-terminus and C-terminus of the sequence in the TANGO plot to reveal the boundaries of the aggregation prone region. (b, e) Site 2 (26 amino acids) or site 3 (25 amino acids) peptides, as well as the indicated mutations of each site, were inserted into the ER-Cλ construct. The sequences were introduced into the flexible Gly/Ser (GS) linker that is downstream of an N-terminal ER-targeting sequence (SS) and followed by mouse $\lambda_1$ light chain constant domain ($C_L$), and ending with a C-terminal, single N-linked glycan acceptor site, NVT, to monitor the translocation of the peptide into the ER lumen. In the case of site 2, three amino acids were altered to lower the aggregation propensity (a), whereas a single amino acid substitution in site 3 was sufficient to reduce propensity to aggregate (d). Since L188 is included in the site 3 sequence, we also made constructs with L188Q. (c, f) 293T cells were transfected with NVT constructs, and 24 hrs later they were metabolically labeled with $^{35}$S methionine and cysteine for 30 minutes. Constructs were immunoprecipitated with anti-λ, treated with (+) or without (-) Endo H, and analyzed by SDS-PAGE to check for glycosylation. The NVT site is only modified (hexagon) upon entry to ER and serves as an indication of full translocation of the peptide sequence into the ER lumen (n=1 for each peptide construct). QVT versions of these constructs were utilized to detect co-chaperone binding in Figure 3. Source data are provided as a Source Data file.

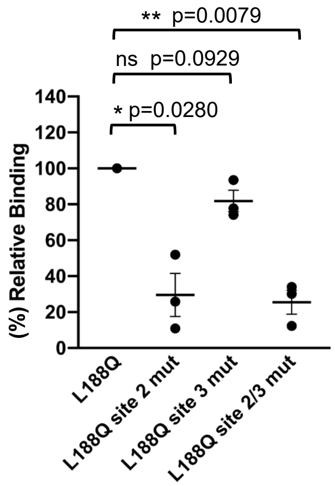


GRP170

| | Mean (Relative to L188Q) | SD | SEM |
|---|---|---|---|
| SPC-L188Q | 100 | 0 | 0 |
| SPC-L188Q site2 mut | 29.6 | 20.8 | 12.0 |
| SPC-L188Q site 3 mut | 81.8 | 10.3 | 5.96 |
| SPC-L188Q site 2/3 mut | 25.5 | 11.5 | 6.67 |

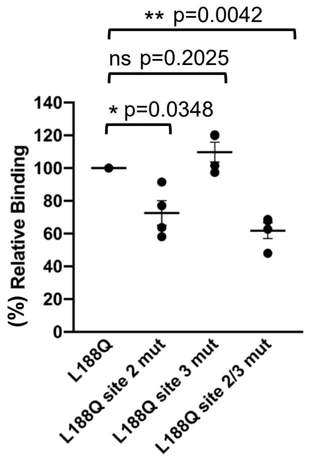


ERdj5

| | Mean (Relative to L188Q) | SD | SEM |
|---|---|---|---|
| SPC-L188Q | 100 | 0 | 0 |
| SPC-L188Q site2 mut | 72.6 | 14.9 | 7.44 |
| SPC-L188Q site 3 mut | 110 | 12.0 | 6.02 |
| SPC-L188Q site 2/3 mut | 61.8 | 9.61 | 4.80 |

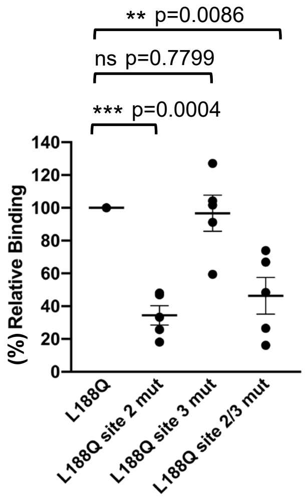


ERdj4

| | Mean (Relative to L188Q) | SD | SEM |
|---|---|---|---|
| SPC-L188Q | 100 | 0 | 0 |
| SPC-L188Q site2 mut | 34.4 | 13.1 | 5.85 |
| SPC-L188Q site 3 mut | 96.7 | 24.6 | 11.0 |
| SPC-L188Q site 2/3 mut | 46.4 | 25.0 | 11.2 |

Supplementary Fig. 4

Supplementary Fig. 4. Quantitation of co-chaperone binding to full-length SP-C L188Q with site 2 and site 3 mutations. Results from immunoprecipitation-coupled western blot experiments shown in Figure 4 were quantified and expressed as a percent of the chaperone that bound to the parental L188Q protein after normalizing for the amount of the client in each case as described in the methods section. Dot plots are shown for each individual experiment done (n=3 for GRP170, n=4 for ERdj5, and n=5 for ERdj4). Horizontal lines and error bars represent the mean and standard error of the mean (SEM), respectively. The difference in co-chaperone binding between SP-C L188Q (parental) and each SP-C mutant (SP-C L188Q site 2, site 3, and site 2/3) was assessed using one-sample *t* test. Source data are provided as a Source Data file.

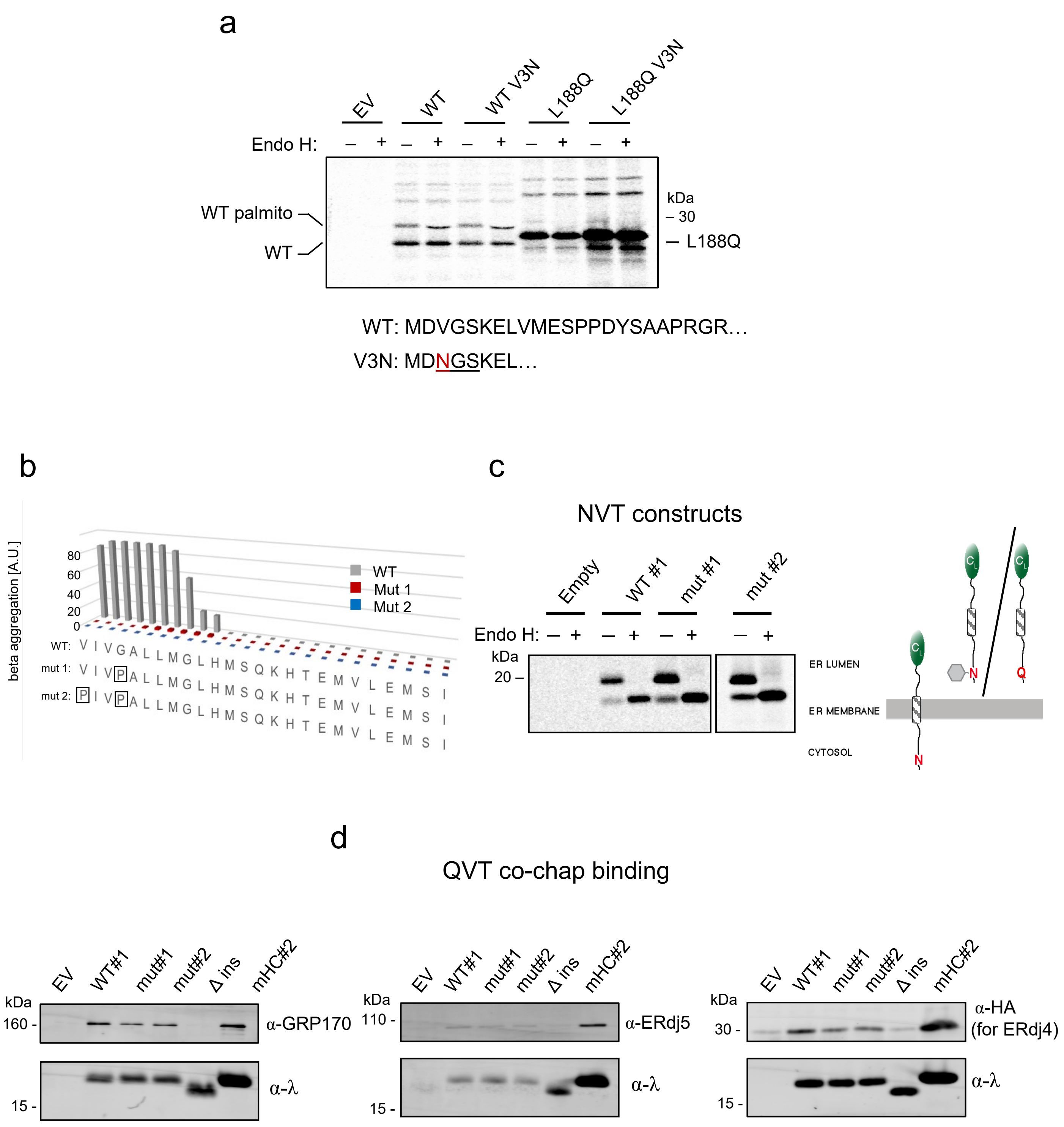


Supplementary Fig. 5

Supplementary Fig. 5. Co-chaperones bind to the region of site 1 that enters the ER lumen. (a) To determine if the entire valine rich region of the L188Q mutant might enter the ER, an acceptor site for N-linked glycosylation was engineered at amino acid 3 by substituting an asparagine for the valine in both the wild-type and L188Q mutant. The versions of wild-type and L188Q without and with the engineered glycan acceptor site were expressed in 293T cells, as was an empty vector (EV). Cells were radiolabeled for 30 min, and proteins were immunoprecipitated with α-SP-C. After isolation, SP-C proteins were divided, one half treated with endo H and the other left untreated, and then both were analyzed by reducing SDS-PAGE (image shown represents n=3 individual experiments). The sequence with the introduced glycan acceptor site is shown below. (b) The 25-amino acid segment containing the portion of site 1 that enters the ER was subjected to the TANGO algorithm. Mutations that reduce aggregation propensity are boxed. (c) 293T cells were transfected with indicated ER-Cλ-NVT constructs. Twenty-four hrs later, cells were metabolically labeled for 30 minutes, and proteins were isolated with anti-λ. Immunoprecipitated proteins were treated with (+) or without (-) Endo H and analyzed by SDS-PAGE (n=1 for WT #1 and mut #1; n=2 for mut #2). (d) ER-Cλ-QVT constructs were co-expressed with the depicted co-chaperones. Lysates were immunoprecipitated with anti-λ, separated on SDS-PAGE gels, and transferred for blotting with the indicated antibodies. A construct with no peptide insert (Δ ins) represented a negative control, and a construct (mHC#2) from original peptide binding study was included as a positive control (images represent n=3 experiments). Source data are provided as a Source Data file.

a

% Glycosylation (n=3)

| | Average | Std. Dev |
|---|---|---|
| WT N67 | 5.6 | 2.8 |
| WT N67+L188Q | 3.4 | 2.8 |

% Palmitoylation (n=5)

| | Average | Std. Dev. |
|---|---|---|
| WT (IP SP-C) | 17.6 | 3.1 |
| WT+3xHA L188Q (IP SP-C) | 10.0 | 2.4 |
| WT+3xHA L188Q (IP HA) | 1.6 | 0.6 |

% Disulfide-Linked L188Q
Oligomers (IP HA) (n=3)

| | Average | Std. Dev. |
|---|---|---|
| 3xHA L188Q | 64 | 1 |
| WT+3xHA L188Q | 54 | 3 |

b

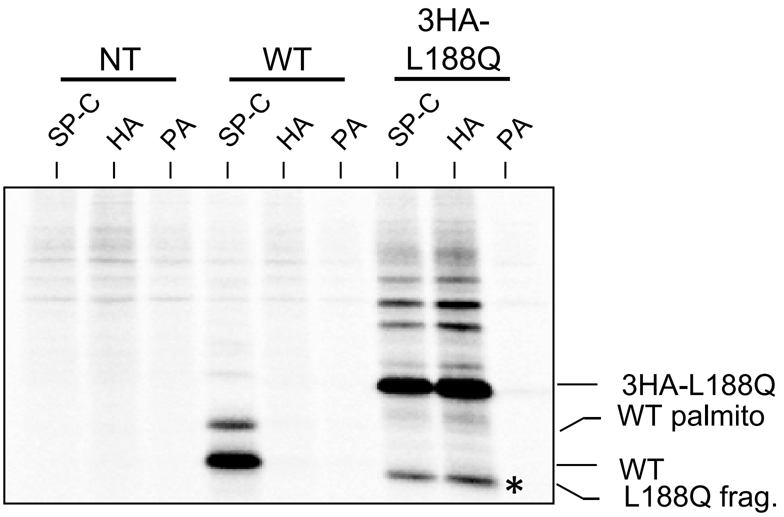


Supplementary Fig. 6

Supplementary Fig.6. Quantification of the effects of co-expressing mutant SP-C protein on wild-type SP-C maturation and demonstration of specificity of immune reagents. (a) A 30-minute radiolabeling coupled with immunoprecipitation was used. The % glycosylation and palmitoylation (data from Figure 6a) of wild-type SP-C with and without 3xHA-L188Q co-expression were calculated after normalizing for levels of wild-type SP-C expression. The effect of wild-type SP-C co-expression on 3xHA-L188Q disulfide-linked oligomers was calculated (data from Figure 6b). The number of biological replicates and standard deviation for each are shown. (b) Cells expressing the depicted constructs were radiolabeled for 30 minutes, and lysates were immunoprecipitated with monoclonal anti-HA, polyclonal SP-C, or Protein A Agarose (PA) beads alone as indicated. Isolated proteins were separated by reducing SDS-PAGE and visualized by phosphorimaging. NT, non-transfected (n=1). These data provide the immunoprecipitation specificity controls for the various constructs used in Figure 6. Source data are provided as a Source Data file.

Supplementary Table 1.  Primer pairs used for mutagenesis

| Site | Forward primer | Reverse primer |
|---|---|---|
| N61<br>SQK→NQT | GACACACACGGAGATGGTTCTG | TGGTTCATGTGGAGACCCATGAG |
| N63<br>KHT→NHT | CATGAGCCAGAACCACACGGAGA<br>T | TGGAGACCCATGAGCAGG |
| N64<br>HTE→NTT | GACGATGGTTCTGGAGATGAGC | GTGTTTTTCTGGCTCATGTGGAG |
| N65<br>TEM→NET | GACGGTTCTGGAGATGAGCATTG | TCGTTGTGTTTCTGGCTCATGTG |
| N67<br>MVL→NVT | TACGGAGATGAGCATTGGGGCG | ACGTTCTCCGTGTGTTTCTGGCTC |
| N68<br>VLE→NLT | GACGATGAGCATTGGGGCGCCG | AGATTCATCTCCGTGTGTTTCTGG<br>CTC |
| N70<br>EMS→NMT | GACCATTGGGGCGCCGGAAGCC | ATGTTCAGAACCATCTCCGTGTG<br>TTTCTGGC |
| Site 1 mut 1 (G52P) | GGTGATTGTGCCAGCCCTGCTCAT<br>G | ACGACGATGAGGACCACC |
| Site 1 mut 2 (G52P, V49P) | GTGCCAGCCCTGCTCATGGGTCTC | AATCGGCACGACGATGAGGACC<br>AC |
| *Site 2 mut | | |
| (F94A) | GTTACCACTGCCACCGCCTCCATC | GATGGAGGCGGTGGCAGTGGTA<br>AC |
| (V102A) | GGCCTCGCGGTGTATGAC | GTCATACACCGCGAGGCC |
| (I111A) | CTGCTGGCCGCCTACAAGC | GCTTGTAGGCGGCCAGCAG |
| *Site 3 mut (F180A) | CCGGCCGCCCTGGGCATG | CATGCCCAGGGCGGCCGG |
| C148S | CAGATGGAATCCTCTCTGCAG | GAAGTTGTGGACTTTTCTATTG |
| N-terminal 3HA tag on L188Q | GTGCCGGATTATGCGTATCCGTAT<br>GATGTGCCGGATTATGCGGATGT<br>GGGCAGCAAAGAG | ATCATACGGATACGCATAATCCG<br>GCACATCATACGGATACGCCATG<br>GTGGCGAATTCGAAG |

**Dpn-I site-directed mutagenesis*
