# Supplementary material for: Mapping SP-C co-chaperone binding sites reveals molecular consequences of disease-causing mutations on protein maturation
Source: Nat Commun. 2022 Apr 5;13:1821. doi: 10.1038/s41467-022-29478-z (PMC8983781; doi:10.1038/s41467-022-29478-z)
Supplement: Supplementary file 3 — Reporting Summary [file 41467_2022_29478_MOESM3_ESM.pdf]

nature portfolio

Corresponding author(s): Linda Hendershot

Last updated by author(s): Feb 22, 2022

Reporting Summary

Nature Portfolio wishes to improve the reproducibility of the work that we publish. This form provides structure for consistency and transparency in reporting. For further information on Nature Portfolio policies, see our Editorial Policies and the Editorial Policy Checklist.

Statistics

For all statistical analyses, confirm that the following items are present in the figure legend, table legend, main text, or Methods section.

n/a	Confirmed	
☐	☒	The exact sample size (*n*) for each experimental group/condition, given as a discrete number and unit of measurement
☐	☒	A statement on whether measurements were taken from distinct samples or whether the same sample was measured repeatedly
☐	☒	The statistical test(s) used AND whether they are one- or two-sided<br>*Only common tests should be described solely by name; describe more complex techniques in the Methods section.*
☒	☐	A description of all covariates tested
☒	☐	A description of any assumptions or corrections, such as tests of normality and adjustment for multiple comparisons
☐	☒	A full description of the statistical parameters including central tendency (e.g. means) or other basic estimates (e.g. regression coefficient) AND variation (e.g. standard deviation) or associated estimates of uncertainty (e.g. confidence intervals)
☐	☒	For null hypothesis testing, the test statistic (e.g. *F*, *t*, *r*) with confidence intervals, effect sizes, degrees of freedom and *P* value noted<br>*Give P values as exact values whenever suitable.*
☒	☐	For Bayesian analysis, information on the choice of priors and Markov chain Monte Carlo settings
☒	☐	For hierarchical and complex designs, identification of the appropriate level for tests and full reporting of outcomes
☒	☐	Estimates of effect sizes (e.g. Cohen's *d*, Pearson's *r*), indicating how they were calculated

Our web collection on statistics for biologists contains articles on many of the points above.

Software and code

Policy information about availability of computer code

Data collection	NA
Data analysis	NA

For manuscripts utilizing custom algorithms or software that are central to the research but not yet described in published literature, software must be made available to editors and reviewers. We strongly encourage code deposition in a community repository (e.g. GitHub). See the Nature Portfolio guidelines for submitting code & software for further information.

Data

Policy information about availability of data

All manuscripts must include a data availability statement. This statement should provide the following information, where applicable:

- Accession codes, unique identifiers, or web links for publicly available datasets
- A description of any restrictions on data availability
- For clinical datasets or third party data, please ensure that the statement adheres to our policy

Primary data for this study are included in the Source Data files provided with this paper.

Field-specific reporting

Please select the one below that is the best fit for your research. If you are not sure, read the appropriate sections before making your selection.

☒ Life sciences ☐ Behavioural & social sciences ☐ Ecological, evolutionary & environmental sciences

For a reference copy of the document with all sections, see nature.com/documents/nr-reporting-summary-flat.pdf

Life sciences study design

All studies must disclose on these points even when the disclosure is negative.

Sample size	No sample size calculations were performed. A per convention in our field, at least three biological replicates were performed for immunoprecipitation and western blotting experiments, except in cases where the SD was larger than the rest. In these cases additional replicates were performed.
Data exclusions	No data was excluded
Replication	Transfection/immunoprecipitation/western blots were performed with at least three biological replicates. In most cases, SD were quite small. In those that were somewhat large, additional replicates were performed.
Randomization	Not relevant to our study, as these experiments were defined by the specific constructs being used.
Blinding	Investigators were not blinded, as samples needed to be loaded on gels in a specific order for comparisons.

Reporting for specific materials, systems and methods

We require information from authors about some types of materials, experimental systems and methods used in many studies. Here, indicate whether each material, system or method listed is relevant to your study. If you are not sure if a list item applies to your research, read the appropriate section before selecting a response.

Materials & experimental systems

n/a	Involved in the study	
☐	☒	Antibodies
☐	☒	Eukaryotic cell lines
☒	☐	Palaeontology and archaeology
☒	☐	Animals and other organisms
☒	☐	Human research participants
☒	☐	Clinical data
☒	☐	Dual use research of concern

Methods

n/a	Involved in the study	
☒	☐	ChIP-seq
☒	☐	Flow cytometry
☒	☐	MRI-based neuroimaging

Antibodies

Antibodies used	All antibodies, their sources with cat #, and dilutions are included in Materials and Methods<br>The rabbit polyclonal anti-GRP170 (1:1000 for western blotting), and mouse monoclonal anti-ERdj4 (0.8ug/ml for western blotting) antibodies were produced in the Hendershot lab. The mouse monoclonal anti-HA antibody was a kind gift from Dr. Al Reynolds (Vanderbilt University, 1:500 for IP, 1:1000 for western blotting). Other antibodies used were obtained commercially and include: goat anti-mouse λ LC (SouthernBiotech, 1060-01, 1:500 for IP,1:1000 for western blotting), HRP-conjugated goat anti-rabbit (Santa Cruz Biotechnology, sc-2054, 1:10,000), HRP-conjugated donkey anti-goat (Santa Cruz Biotechnology, sc-2020, 1:10,000), HRP-conjugated goat anti-mouse (SouthernBiotech, 1038-05, 1:10,000), monoclonal anti-ERdj5 (Abnova, H00054431-M01, 1:1,000 for western blotting), polyclonal rabbit anti-pro-SP-C (Seven Hills Bioreagents, WRAB9337, rabbit bleed 364, 1:2,000 for IP, 1:5000 for western blotting), mouse monoclonal anti-β-Actin (Sigma-Aldrich, AC-15, 1:2,000 for western blotting), and IRdye® secondary antibodies (LI-COR Biosciences, all 1:20,000): goat anti-mouse IgG (925-32210), goat anti-rabbit IgG (925-68071), and donkey anti-goat IgG (926-68074).
Validation	Data provided in the manuscript

Eukaryotic cell lines

Policy information about cell lines

Cell line source(s)	293T cell line and its source from ATCC is included in Materials and Methods
Authentication	293T cells used in this study and have been authenticated.

Mycoplasma contamination

All lines are tested for mycoplasma before making frozen aliquots and thawed cells are used for only 6 or fewer weeks.

Commonly misidentified lines
(See ICLAC register)

No commonly misidentified cell lines were detected or used.
